# iBio-GATS—A Semi-Automated Workflow for Structural Modelling of Insect Odorant Receptors

**DOI:** 10.3390/ijms25053055

**Published:** 2024-03-06

**Authors:** Vaanathi Chidambara Thanu, Amara Jabeen, Shoba Ranganathan

**Affiliations:** Applied Biosciences, Macquarie University, Sydney 2109, Australia; vaanathi.chidambarathanu@students.mq.edu.au

**Keywords:** insect odorant receptor, seven transmembrane helical protein, template-based modelling, semi-automated workflow, 3D structural model, membrane protein

## Abstract

Insects utilize seven transmembrane (7TM) odorant receptor (iOR) proteins, with an inverted topology compared to G-protein coupled receptors (GPCRs), to detect chemical cues in the environment. For pest biocontrol, chemical attractants are used to trap insect pests. However, with the influx of invasive insect pests, novel odorants are urgently needed, specifically designed to match 3D iOR structures. Experimental structural determination of these membrane receptors remains challenging and only four experimental iOR structures from two evolutionarily distant organisms have been solved. Template-based modelling (TBM) is a complementary approach, to generate model structures, selecting templates based on sequence identity. As the iOR family is highly divergent, a different template selection approach than sequence identity is needed. Bio-GATS template selection for GPCRs, based on hydrophobicity correspondence, has been morphed into iBio-GATS, for template selection from available experimental iOR structures. This easy-to-use semi-automated workflow has been extended to generate high-quality models from any iOR sequence from the selected template, using Python and shell scripting. This workflow was successfully validated on *Apocrypta bakeri* Orco and *Machilis hrabei* OR5 structures. iBio-GATS models generated for the fruit fly iOR, OR59b and Orco, yielded functional ligand binding results concordant with experimental mutagenesis findings, compared to AlphaFold2 models.

## 1. Introduction

Insect pests pose a significant influence on both agricultural production and food availability. A primary factor contributing to diminished agricultural yields is the detrimental impact inflicted by these pests. Globally, insect pests are estimated to be responsible for an annual reduction of 18–20% [1] in overall agricultural output. The rise in insecticide resistance has prompted a shift towards environmentally friendly strategies, such as the use of attractants and repellents, which are now increasingly prevalent. For example, the Queensland fruit fly, *Bactrocera tryoni*, a major pest affecting fruits and vegetables in Australia, has been found to be strongly attracted to a synthetic raspberry-ketone analogue, cuelure (4-(4-acetoxyphenyl)-2-butanone) [2], commercially used in traps (https://biotrap.com.au/products/attractants/ft-cuelure/; accessed on 20 December 2023).

The sense of olfaction plays a crucial role for insects as it enables them to detect essential chemical cues in their surroundings, facilitating activities such as locating food and mates, among other vital actions [3]. Over the past two decades, extensive research has been conducted on olfaction in insects. The ongoing investigation into the mechanisms of insect olfaction holds promise for advancing the development of insect repellents that are both more efficient and environmentally friendly for the management of insect pests. A recent review on insect pest control has emphasized the importance of odorant receptors within the insect olfactory system [4].

Insects possess highly sensitive receptors that enable them to differentiate between a vast array of odorants. Insect odorant receptors (iORs) are members of the family of the seven transmembrane helix (7TM) proteins, that form heteromeric ligand-gated cation channels, which composed of ligand-specific receptor and the co-receptor (Orco) [5]. The sequence similarity among iORs is low, with a maximum identity of about 20% [6]. The number of known iORs ranges from 37 to 180, leading to the development of insectOR, for accurate prediction of iOR genes from newly sequenced genomes [7].

**Figure 1:**
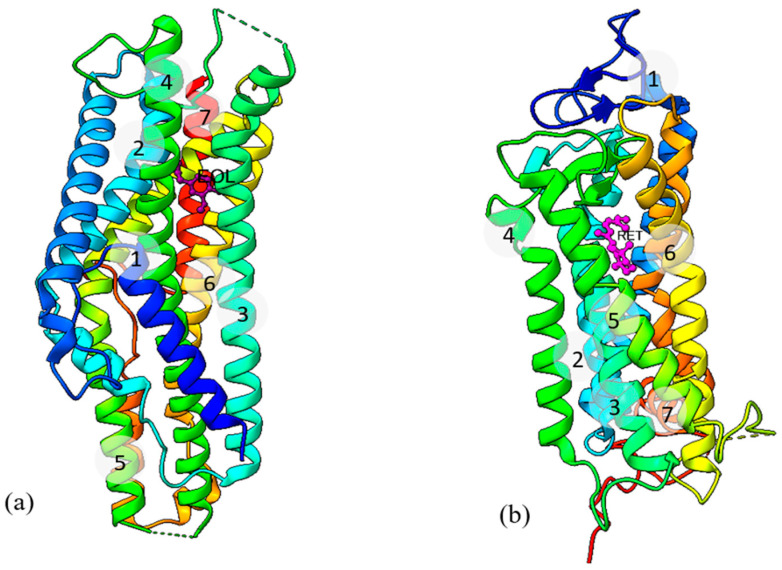
(**a**) Structure of *Mh*OR5 with bound eugenol (EOL, shown in purple, PDB ID: 7LID and (**b**) Structure of bovine rhodopsin with bound retinal (RET, shown in pink) (PDB ID:1F88) [12]. The structures are presented in cartoon representation with rainbow colouring, from blue (N-terminus) to red (C-terminus).

iORs are similar to G-protein coupled receptors (GPCRs) but exhibit an inverted topology [8] with an intracellular N-terminus and an extracellular C-terminus. Also, they lack the conserved motifs found in GPCRs [9]. Similar to the vertebrate olfactory system, the insect olfactory system also exhibits good sensitivity to specific odorants. As these iORs are membrane proteins that require a phospholipid bilayer to function, experimental structural investigation is difficult, challenged by loop flexibility, low stability, and partially hydrophobic surface. Recently, cryo-electron microscopy (cryo-EM) structures of iORs were deposited in the Protein Data Bank (PDB; https://www.rcsb.org; accessed on 15 August 2023) from the Ruta lab [10,11] and. Both studies revealed a tetrameric structure with a functional channel pore. The 2018 study [10] was on the homomeric yet functional Orco structure of the wasp, *Apocrypta bakeri* (*Ab*Orco; PDB ID: 6C70), with a cation channel that is activated by the agonist VUAA1 (*N*-(4-Ethylphenyl)-2-{[4-ethyl-5-(pyridin-3-yl)-4*H*-1,2,4-triazol-3-yl]sulfanyl}acetamide) under laboratory conditions. Following this, the 2021 structure of the homomeric *Mh*OR5 receptor from the jumping bristletail, *Machilis hrabei*, which functions without Orco, was solved. Three *Mh*OR5 structures are available: without any ligand (*apo* form; PDB ID: 7LIC), and with two bound agonistic ligands, eugenol (PDB ID: 7LID) and the insect repellent DEET (N, N-diethyl-meta-toluamide) (PDB ID: 7LIG). Both odorant molecules were bound at the same location in the receptor, located ~15 Å deep within the transmembrane region of each subunit offering a structural basis for the diverse chemical sensitivity of iORs. This intermembrane location of the bound ligands is similar to the GPCR ligand binding pocket, e.g., in bovine rhodopsin [12] (Figure 1, using ChimeraX 1.4 [13]), despite the inverted topology. 

Protein structure prediction uses computational methods to deduce a protein’s three-dimensional structure. Given the difficulties in experimentally determining iOR structures, computational methods of predicting structural 3D models can be a reliable alternative to experimental methods for the structure prediction of these ORs. 

Protein structures can be predicted by template-based and template-free (ab initio or free modelling) approaches. Template-based modelling (TBM), based on the identification and alignment of a related protein with a known structure, is more accurate than template-free modelling, which focuses on fold prediction from physics-based energy functions [14]. Also known as homology or comparative modelling [15], TBM involves identifying homologues with experimental structures from the PDB, followed by aligning the target sequence to that of the template structure. Modeller [16], one of the best model building tools available, can then be used to build a 3D structural model. However, when sequence conservation is low within a protein family, e.g., GPCRs, mapping the target sequence to the template, requires considerable attention. Despite low sequence identity, hydrophobicity patterns in the TM regions of GPCRs are similar, leading to structural homology [17]. To select structural templates for GPCR sequences, Jabeen et al. [18] have proposed a biophysical approach, called Bio-GATS, based on hydrophobicity correspondence (HC), resolution, hotspot residues, and target coverage for template selection. Bio-GATS has been applied to the human olfactory receptor, OR1A1, to develop a structural model using comparative modelling that could explain experimental mutagenesis data against six ligands. While Bio-GATS uses BLAST for the initial alignment, iORs do not have any conserved motifs or patterns that can assist with BLAST alignment. For such membrane proteins, AlignME [19] integrates membrane-specific information such as hydrophobicity scales to create an optimal alignment. 

Several automated template-free structure modelling (FM) programs are available now. The application of deep machine learning (AlphaFold (v.2.0) [20], iTASSER [21], Rosetta [22]) to the prediction process has transformed the field of homology modelling in recent years, greatly enhancing model generation [23]. A recent compilation of AlphaFold2 models for a number of iORs is available from iORBase [24]. Also, a complete collection of AlphaFold2 models for all protein sequences is available from the AlphaFold database [AlphaFold DB, https://alphafold.ebi.ac.uk; accessed on 27 January 2024]. However, as the structures in iORBase and AlphaFold DB are only computational models, they are not suitable as structural templates. Moreover, such sources only address part of the structure prediction problem, as these databases do not provide any models with mutations or post-translational modifications that affect protein structure and function [25]. Specifically, when the AlphaFold2 models for aphid odorant receptors were compared to TBM results, certain key residues in the ligand binding pocket were lacking [26]. 

Here we present an easy-to-use interactive semi-automatic workflow, based on the biophysical approach of hydrophobicity correspondence [18], for generating high-quality models of iORs. This workflow was developed by adapting the Python-based tool Bio-GATS to accommodate iOR structures with inverted topology (iBio-GATS) and then by integrating Modeller for TBM; through shell scripting, to rapidly generate high-quality iOR models. Users have the flexibility to execute the workflow either in a fully automated mode or by selecting a preferred template. iBio-GATS workflow can rapidly generate high-quality structural models of iORs for any pest species in a high-throughput fashion. 

Even when there are currently only two iORs with structures, the accuracy of iBio-GATS template selection and alignment far exceeds traditional sequence-based template identification and alignment methods, from our successful validation studies, using the sequence of *Ab*Orco and *Mh*OR5 in turn as query sequences. We have applied iBio-GATS to the vinegar fruit fly *Drosophila melanogaster* OR, *Dm*OR59b (UniProt ID: Q9W1P8), with experimental results for the naturally occurring mutant [27] for DEET binding and *Dmel*Orco (UniProt ID: Q9VNB5, as referred to by Pacalon et al. [28]) with experimental mutagenesis data for VUAA1 binding and then compared our results to AlphaFold2 models.

## 2. Results and Discussion

Details of data sources and computational methods are available in Section 3, with only brief descriptions and essential references cited below.

### 2.1. iBio-GATS Workflow Development

**Figure 2:**
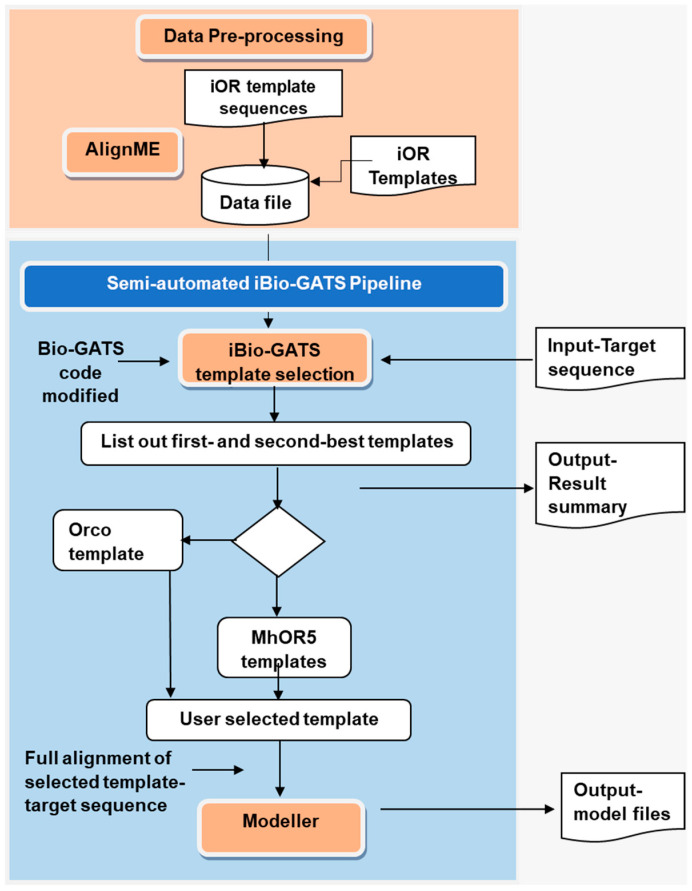
Flowchart of the iBio-GATS workflow showing the process flow and program integration.

The steps involved in the workflow development are shown in Figure 2, using bash scripting, a commonly employed technique in automation [29], to integrate programs. 

#### 2.1.1. Data Pre-Processing

The input data for iBio-GATS consists of all available iOR template structures (one for *Ab*Orco and three for *Mh*OR5) and the two sequences corresponding to these templates. For each sequence, AlignMe 1.2 [19] was used to define the positions of the seven TM helices, which is crucial for model building. The centre residue of each defined helix serves as anchoring point for aligning the target and template as unlike GPCRs, there are no known conserved motifs for iORs. iOR template sequences with their helix definitions and centre residues (pre-processed data) were entered into an Excel sheet which is required to be supplied for workflow execution. 

#### 2.1.2. Computational Workflow

**Figure 3:**
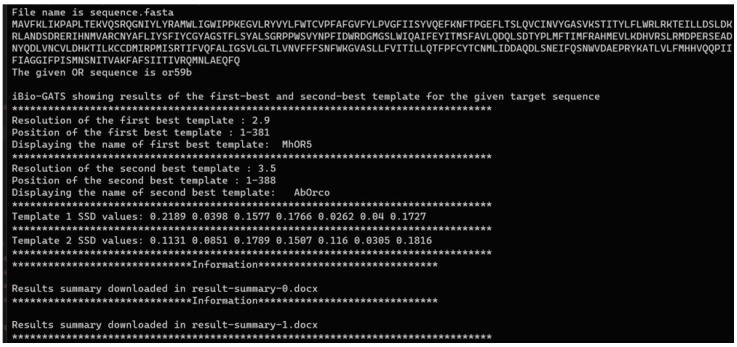
Template selection output summary. The name, resolution, and SSD values for each of the seven helices, for both the template sequences, are displayed on the command line window in the workflow. *Dm*OR59b (UniProt ID: Q9W1P8) is supplied as an input sequence for illustration.

- Template selection: The target iOR sequence is input to iBio-GATS, for determining the top templates, based on the alignment of TM helical segments using AlignMe, followed by the calculation of hydrophobicity correspondence for each helix using the sum of squared differences (SSD) values (details available in [18]). The user is given the option of selecting the preferred template, particularly as *Mh*OR5 has three structures, with the same sequence. The user may also check and modify the full alignment generated by iBio-GATS, to ensure the TM segments are gap-free. The output from this step is shown in Figure 3. User can also edit the helix-wise target-template alignment based on SSD calculations.
- Model building: The TBM program, Modeller will execute automatically upon the selection of the template for the target sequence, based on the template-target pair alignment provided, the target sequence, and the PDB structure file of the selected template. The output consists of five predicted models.

### 2.2. iBio-GATS Validation

We conducted a benchmarking study to validate the workflow using the iOR structural templates. This is done by applying the workflow to the experimental iOR templates (PDB IDs: 6C70 and 7LIG) by using them interchangeably to perform cross-validation. Despite the sequence identity of the iOR structural templates (*Mh*OR5 and *Ab*Orco) being low (17.6%), hydrophobicity patterns in the helices are similar, which results in structural homology. For both targets, their cognate structures were selected as the top template by iBio-GATS, as expected, but we selected the second-best template (i.e., the “other” structure) was selected for building models for the target sequence. Thus, for *Mh*OR5, *Ab*Orco was selected as a template, and for *Ab*Orco, *Mh*OR5 was selected for cross-validation.

The hydrophobicity correspondence for the *Ab*Orco-*Mh*OR5 pair, measured in SSD values [18], is available in Table 1. 

Hydrophobicity plots, helical plots, seven helical alignments, and the full alignment generated for the *Ab*Orco target using the template *Mh*OR5, are available in Supplementary Figures S1–S4. The results for *Mh*OR5 as target with *Ab*Orco as template were very similar to the SSD values for all seven helices for the above *Mh*OR5 template—*Ab*Orco target pair are listed in Table 1. Analysing all the hydrophobicity plots (Supplementary Figure S1) for one of the target-template pair (*Ab*Orco with its template *Mh*OR5), the two graphs are similar with low SSD values, showing good hydrophobicity correspondence (HC) for all the seven helices. The helical wheel plot (Supplementary Figure S2) for the same target-template pair (*Ab*Orco with its template *Mh*OR5) shows hydrophobic moments for the majority of helices pointing in the same direction, except for helices 2 and 3 (with values close to zero). From the seven helical alignments (Supplementary Figure S3), the full alignment for the *Ab*Orco target with the template *Mh*OR5 (Supplementary Figure S4), was generated for model building. Out of the five models generated, the best model was selected based on the lowest Modeller objective function. The models predicted by the workflow for both template sequences are shown in Supplementary Figure S5.

**Figure 4:**
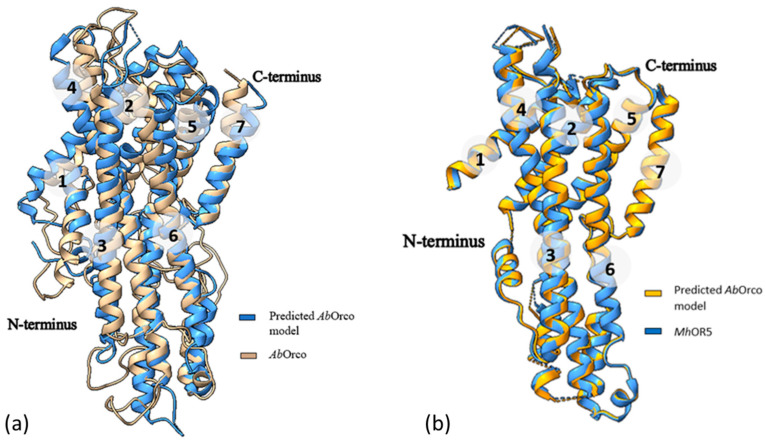
Superimposition of the predicted model of *Ab*Orco with (**a**) its experimental PDB structure (PDB ID: 6C70), with an RMSD of 1.13 Å and (**b**) its template *Mh*OR5 showing only the helical regions, with an RMSD of 0.72 Å.

We then proceeded to evaluate the accuracy of the predicted models for *Ab*Orco and *Mh*OR5 by calculating the RMSD (root mean square deviation) against cognate experimental structures. For *Ab*Orco, the RMSD between the predicted model and the experimental structure (PDB ID: 6C70) was 1.13 Å (Figure 4a) for the full model, with an RMSD of 0.72 Å for helical region as determined using ChimeraX [12]. Similarly, for *Mh*OR5, the RMSD between the predicted model and its experimental structure (PDB ID: 7LIG) was 1.23 Å for the full model and 1.07 Å (Figure 4b) for the helical region. Additionally, the RMSD between the predicted model of *Mh*OR5 and its template, *Ab*Orco (PDB ID: 6C70) was found to be 0.56 Å. 

The superimposed structures for both the predicted models were found to be very close, aligning all the seven helices structurally with their cognate structures, with some differences only within the loop regions. The RMSD obtained by comparing the models completely with their cognate experimental structures was ~1 Å, showing the accuracy of the models generated by this approach. The workflow developed is thus generic, and can be applied to any iOR sequence for rapidly generating high-quality structural models.

### 2.3. Application to the Vinegar Fruit Fly OR, DmOR59b

iBio-GATS was used to model a vinegar fruit fly *Drosophila melanogaster* OR, *Dm*OR59b (UniProt ID: Q9W1P8), with experimental mutagenesis data. This OR was selected as a case study, because *D. melanogaster* is a well-characterized model organism with known OR repertoire and has been studied experimentally. The predicted structure was validated based on the experimental mutational data [27], where a single amino acid polymorphism, V91A, in the second helix of *Dm*OR59b altered the sensitivity of the receptor to DEET, making the receptor inactive to DEET-induced modulation.

#### 2.3.1. MhOR5 Is the Best Template for the DmOR59b Sequence

The workflow starts by defining the position of each helix in the target sequence *Dm*OR59b, based on the pairwise alignment with each iOR template using AlignMe. The pairwise alignment of the target was also repeated with the other template *Ab*Orco. Subsequently, the positions of the helices and the central residues (Supplementary Table S1) identified for the target sequence were recorded and added to the iOR data file in iBio-GATS. Details of helix definition are provided in the example file for user reference in the GitHub repository (https://github.com/CVaans/iBio-GATS; accessed on 5 August 2023). The *Dm*OR59b target sequence was provided as input for the workflow, generating result summaries for both template sequences. *Mh*OR5 was selected as the first-best template by the workflow, with a pairwise sequence identity of only 12.9%. Table 2 lists the SSD values for all seven helices of *Dm*OR59b against both templates. 

**Figure 5:**
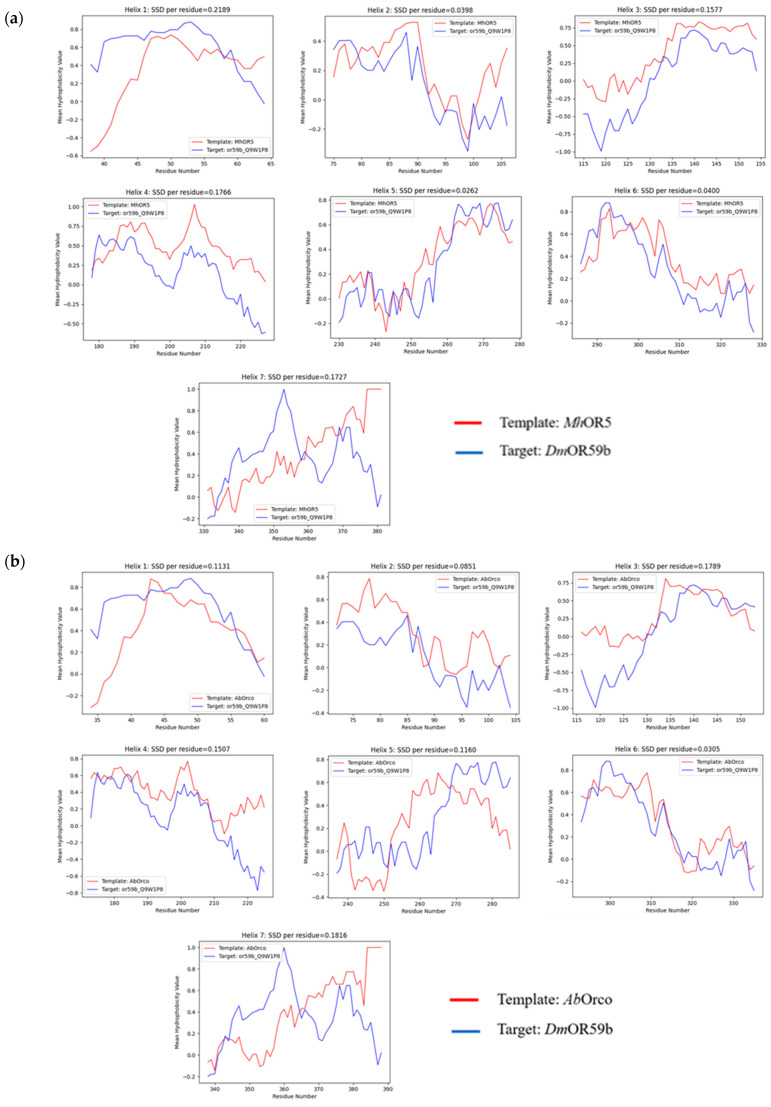
Hydrophobicity plots for all the seven helices for the target sequence, *Dm*OR59b with the templates (**a**) *Mh*OR5 and (**b**) *Ab*Orco.

Figure 5 provides the hydrophobicity plots generated for *Dm*OR59b with both the first- and second-best templates. *Dm*OR59b showed good HC for all the helices except 1 and 7 with *Mh*OR5 while it showed good HC for the helices 2,4 and 6 but not for helices 1, 5 and 7 with *Ab*Orco. Comparing the helical wheel plots (Supplementary Figure S6) for the templates, helices 2, 4, and 7 of *Dm*OR59b are more amphiphilic than other helices. The hydrophobic moments for all helices of *Dm*OR59b except helix 4 are closely aligned to those of *Mh*OR5 while the hydrophobic moments of *Dm*OR59b for the helices 4, 5, 6 and 7 are pointing almost in the same direction as those of *Ab*Orco. Overall, *Mh*OR5 showed a better score for hydrophobicity correspondence for the target *Dm*OR59b, than *Ab*Orco. 

The full alignment generated by iBio-GATS for *Dm*OR59b with *Mh*OR5 was manually edited to minimise the gaps in the helical segments, with MEGA11 [30] (Supplementary Figure S7). The edited alignment of *Dm*OR59b and *Mh*OR5 was input to Modeller, to generate model structures. Of the three structures available for *Mh*OR5, the one with DEET (PDB ID:7LIG) was selected to generate the workflow model (Supplementary Figure S8a), since the mutant data was for DEET binding, although the ligand is not present in the template structure provided for model building (see Section 3.1.2). To ensure this selection did not introduce any bias, models were also generated the other two *Mh*OR5 structures, 7LIC (*apo*) and 7LID (with bound eugenol). The two new models were overlaid with the model generated from 7LIG (Supplementary Figure S8b) and the pairwise RMSD was found to be <1.0 Å, suggesting that any of the three *Mh*OR5 templates may be selected for a query iOR sequence. 

Based on the naturally occurring mutant in the *Dm*OR59b sequence, a single amino acid polymorphism (V91A) was introduced into the sequence using MEGA11, and the best model generated using the workflow for this mutant sequence using the same 7LIG template. The models predicted are referred to as wild-type (Wt) and mutant (Mt) models. To compare with AlphaFold 2 (Af) models, the Wt *Dm*OR59b model was downloaded from the AlphaFold database, while the Mt *Dm*OR59b model was generated using the AlphaFold2 workflow tool of Galaxy Australia (https://usegalaxy.org.au/; accessed on 28 January 2024) [31]. The ligand DEET, downloaded from PubChem database (https://pubchem.ncbi.nlm.nih.gov/; accessed on 25 January 2024); with Compound ID (CID) 4284) was then docked to the workflow (Wf) models of Wt and Mt *Dm*OR59b (Supplementary Figure S8) and compared with the corresponding Af models. 

#### 2.3.2. Model Quality Analysis

**Figure 6:**
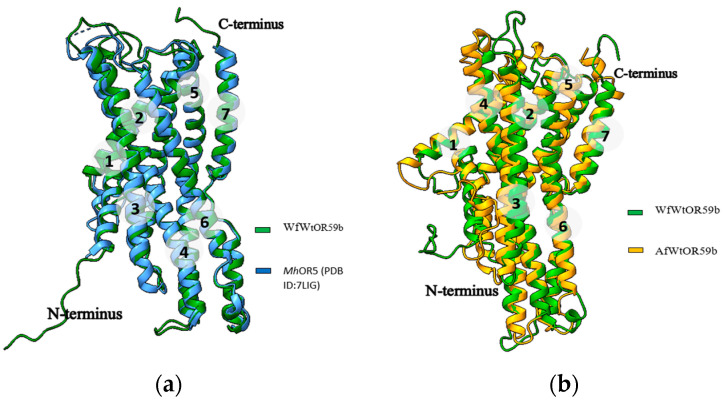
Superimposition of the predicted WfWtOR59b with (**a**) the template *Mh*OR5 (**b**) the AfWtOR59b model.

Superimposing the predicted WfWtOR59b model and its template *Mh*OR5 (PDB ID:7LIG) is shown in Figure 6a. The RMSD was found to be only 0.56 Å. The AfWtOR59b model was superimposed with the same structure with an RMSD of 1.10 Å. To compare the AfWtOR59b model and WfWtOR59b, both were superimposed (Figure 6b) and the RMSD was found to be 1.17 Å which shows the models are not very different.

Structure quality analysis of the predicted models was assessed using PSVS (Protein Structure Validation Software Suite) [32] (https://montelionelab.chem.rpi.edu/PSVS; accessed on 28 January 2024) and the structure quality analysis program, PROSA [33] (https://prosa.services.came.sbg.ac.at/prosa.php; accessed on 28 January 2024) determines how much a structure’s total energy deviates from an energy distribution generated from random conformations. The results are presented in Table 3. Analysing the WfWtOR59b model, 93.9% of the residues are in favourable regions and 6.0% are in allowed regions of the Ramachandran plot computed, with no amino acids in the disallowed regions. The AfWtOR59b model has better results, with 97.2% of the residues are in favourable regions and 2.8% are in allowed regions and no amino acids detected in the disallowed regions (Supplementary Figure S9). RMSD values for bond lengths and bond angles for both models were excellent (less than 0.02 Å and 4.00°, respectively), along with PROSA scores < −3.5, defining high quality structures [34]. 

#### 2.3.3. Ligand Docking

**Figure 7:**
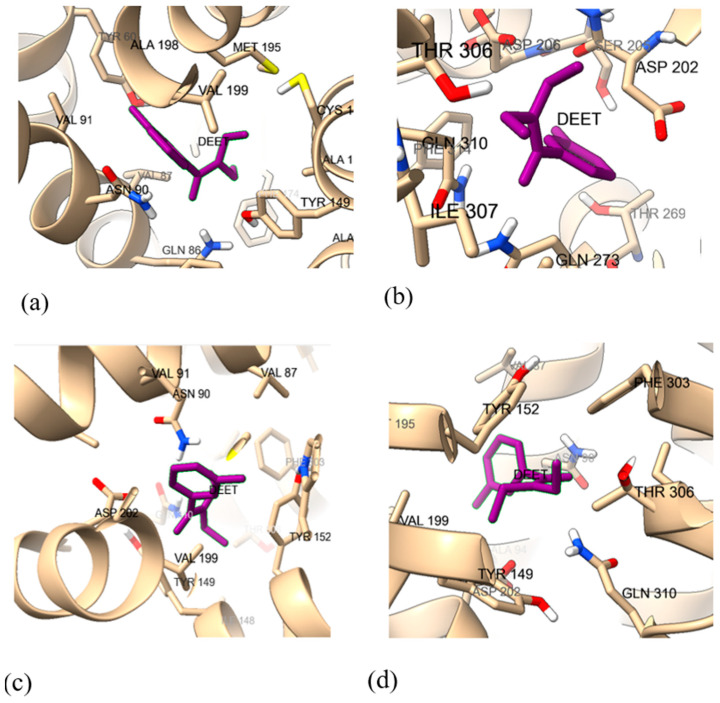
The binding pocket residues of *Dm*OR59b models with bound DEET for (**a**) WfWtOR59b, (**b**) WfMtOR59b, (**c**) AfWtOR59b (binding Pocket 2) and (**d**) AfMtOR59b. V91 is boxed in red.

DEET was docked to the predicted Wf and Af models of wild-type and mutant *Dm*OR59b using ICM-pro 3.9 [35], to check if DEET bound to the predicted models and to verify if V91 was interacting with DEET in the binding pocket, as shown in Figure 7. Pocket positions are available in Supplementary Figure S10. The exterior ligand binding pocket (pocket 1) of the Af model (Supplementary Figure S10c) was not considered and the interior pocket (pocket 2), located in proximity to *Mh*OR5′s ligand binding pocket was selected for docking. V91 interacts with DEET in both Wf and Af wild-type models, unlike the V91A mutant models. DEET is in the binding pocket of all four models and the binding free energy value for each complex is evaluated from molecular dynamics simulation results, described the in following section.

#### 2.3.4. Molecular Dynamics Simulations

To check the robustness of ligand binding and to compute binding free energy values, molecular dynamics simulations were performed in a membrane environment (Details are available in Section 3) for each protein-ligand complex of the predicted models, using AMBER 22 [36]. The length of the simulation used in this study is 100 ns, a time frame comparable to those employed in other recent ligand binding molecular dynamics simulation studies on *Mh*OR5 (70 ns) [37] and *Aedes aegypti* (50 ns) [38]. For ligand channel dynamics, the 500 ns molecular dynamics study of Pacalon et al. [28] would be more appropriate. The ligand-binding free energy values (ΔG_bind_) for DEET were computed by the MM/PBSA (Poisson–Boltzmann) method. The results support the WfWtOR59b model (−4.45 kcal mol^−1^), compared to AfWtOR59b in which DEET is a non-binder (3.52 kcal mol^−1^). Also, the binding affinity of the Wf model is greatly reduced by the V91A mutation (binding energy increased from −4.45 kcal mol^−1^ to 3.15 kcal mol^−1^), with DEET changing from Wt binder to Mt non-binder. On the other hand, the Af model is unable to capture this difference (binding energy reduced from 3.52 kcal mol^−1^ to 1.58 kcal mol^−1^), with the mutant showing increased ligand binding affinity, although both Af models are non-binders.

**Figure 8:**
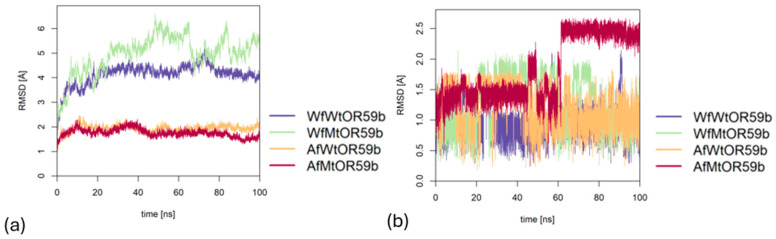
RMSD plots of all four *Dm*OR59b complexes over 100 ns of MD simulations, tracking (**a**) the entire complex and (**b**) the ligand DEET.

Trajectories generated during the MD simulations were analyzed for all four ligand bound models, with the help of CPPTRAJ [39] module of Amber 22 (shown in Figure 8).

The complexes of DEET with both AlphaFold2 models, AfWtOR59b and AfMtOR59b, were comparatively stable throughout 100 ns, suggesting that the V91A mutation had no effect on the structure (Figure 8a). On the contrary, DEET complexes with the iBio-GATS models, WfWtOR59b and WfMtOR59b, (Figure 8a) show that the Wt complex is more stable than the mutant complex, possibly explaining the experimental perturbation of the mutant for DEET binding. Moreover, comparing the two wild-type models, DEET was better stabilized within the binding pocket of the WfWtOR59b model than in the AfWtOR59b model as indicated in Figure 8b. Additionally, comparing the iBio-GATS models, DEET was not stabilized in the mutant complex with WfMtOR59b, which supports the experimental result that the mutation has altered the sensitivity of the *Dm*OR59b towards DEET. In the case of the Af models, the MD simulations were unable to distinguish the binding of DEET to both the Wt and the mutant models, as the DEET position is highly variable and therefore, not stabilized, despite the complex RMSD showing limited fluctuation, possibly dampened by the large receptor protein. The standard deviation (SD) of DEET within the binding pocket of the AfWtOR59b was 0.21 Å compared to 0.35 Å in the WfWtOR59b model while for the AfMtOR59b model, the SD for the last 10 ns was 0.10Å compared to 0.22 Å in WfMtOR59b model.

We also applied our workflow to *D. melanogaster* Orco (Uniprot ID: Q9VNB5), for which a number of mutant structures were functionally evaluated [28]. Insect Orco sequences are highly conserved and *Dmel*Orco (using the abbreviation of Pacalon et al. [28]) and *Ab*Orco sequences are 55.2% identical (Supplementary Figure S11). The centre residue of each helix in *Dmel*Orco was identified (Supplementary Table S2) as the first step in the workflow. iBio-GATS then picked *Ab*Orco as the best template for *Dmel*Orco (Supplementary Figures S12 and S13), as expected. The predicted wild-type model, WfWt*Dmel*Orco, has an RMSD of only 0.1 Å in the helical region, compared to the template structure of *Ab*Orco, with all five VUAA1 binding residues conserved (Supplementary Figure S14). 

**Figure 9:**
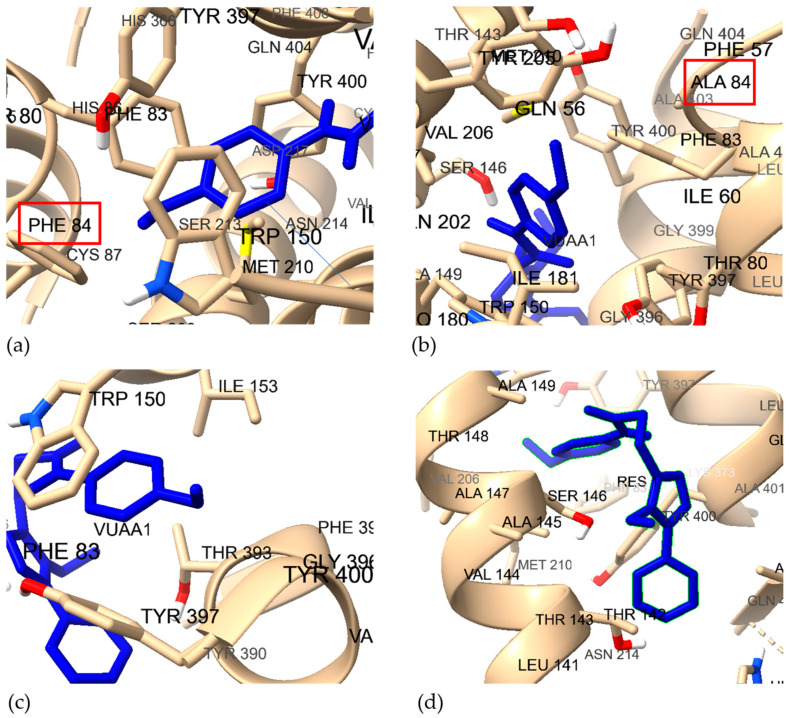
The binding pocket residues of *Dmel*Orco models with bound VUAA1, for (**a**) WfWt*Dmel*Orco, (**b**) WfMt*Dmel*Orco, (**c**) AfWt*Dmel*Orco and (**d**) AfMt*Dmel*Orco. The experimentally mutated residue 84 (Phe84/Ala84) is boxed in red.

We then generated the F84A mutant model (WfMt*Dmel*Orco) and the corresponding AlphaFold2 wild type and mutant models (AfWt*Dmel*Orco and AfMt*Dmel*Orco, respectively), using the same procedures detailed above for *Dm*OR59b. The residues involved in binding VUAA1 are shown in Figure 9. The binding site for the Af models is lacking residue 84, whereas F84/A84 are captured in the Wf models.

The molecular dynamics simulation trajectory analysis over 100 ns provided in the Supplementary Figure S15. The results for the RMSD plots for the four complexes are remarkably similar to those of *Dm*OR59b, the mutation having very little effect on the Af models, possibly due to the lack of contribution from residue 84 to the binding pocket (Figure 9). On the other hand, the wild type Wf model is much more stable than the mutant Wf model. Also, the ligand RMSD values are very similar for the Af models, while the ligand shows greater fluctuation with higher RMSD values for the Wf models, with the mutant model showing the highest RMSD values. 

The binding affinity of the Wf model was greatly reduced after incorporating F84A mutation with the ligand-binding free energy values (ΔG_bind_) value increasing from −4.89 kcal mol^−1^ (binding) to 1.86 kcal mol^−1^ (non-binding) while the Af model was unable to capture this difference, with VUAA1 binding better to the mutant model (binding free energy reduced from −3.50 kcal mol^−1^ to −5.82 kcal mol^−1^). Our study demonstrated that the F84A mutation significantly attenuated VUAA1 responses in *Dmel*Orco.

In summary, MD simulations provided binding free energy values from the iBio-GATS models in accord with the experimental results of Pellegrino et al. [27] for *Dm*OR59b and with the recent mutagenesis studies of Pacalon et al. [28] for *Dmel*Orco. Our workflow is able to generate accurate high-quality functional 3D models for membrane-bound iORs. The results from AlphaFold2 support the identification of the overall fold, but lack the functional binding site for iORs and are unable to support experimental mutational data, as also noted for aphid odorant receptors [26].

## 3. Materials and Methods

### 3.1. Input Data

#### 3.1.1. Target iOR Sequence

The workflow begins by providing as input, the target iOR sequence, representing any iOR sequence of interest for which a structural model needs to be generated. The iOR sequence used in this study, *Dm*OR59b from *Drosophila melanogaster* was acquired from the UniProt database (https://www.uniprot.org/; accessed on 21 January 2024) with the UniProt ID: Q9W1P8 and NCBI Reference Sequence: NP_523822.1. The other sequence used in this study is *Dmel*Orco from the same organism and was also acquired from the UniProt database with UniProt ID: Q9VNB5.

#### 3.1.2. Template iOR Structures from the Protein Data Bank (PDB)

The available experimental iOR structures were given as input templates (listed in Table 4). These structures, downloaded from PDB, were used as templates for template-based modelling of iOR sequences in this study. The PDB file of each template contains four chains and only one chain (Chain A) was retained as template. Also, the bound ligand molecules were deleted.

iBio-GATS input requires the structure-derived sequence from the PDB file of each template (Supplementary Figure S16). PDB structures do not always have coordinates for all residues in their protein sequences. The PDB sequence was extracted using BIOVIA Discovery Studio Visualizer (version 21.1.0.20298) [40] and saved as a separate file, for each structure and added to the “Templates” sheet of the iOR datafile.

### 3.2. Pairwise Sequence Alignment and Helices Definition Extraction

The helical positions of the templates (Supplementary Tables S2 and S3) were derived from the experimental structures [10,11], and entered into a datafile, as input to iBio-GATS. The seven helices of the target sequence were defined by conducting pairwise sequence alignments between each template and the target sequence using the AlignMe program [19]. AlignMe is a pairwise sequence alignment program primarily designed for membrane proteins. It incorporates membrane-specific information, such as transmembrane predictions, hydrophobicity scales, and secondary structure predictions. In our study, AlignMe PS, configured with default parameters, was utilized.

### 3.3. Template Selection Modification in iBio-GATS

Bio-GATS [18], designed as an automated template selection approach for human GPCRs, was adapted for selecting templates for iORs in this study. While the original Bio-GATS method relies on biophysical parameters, including hydrophobicity correspondence, resolution, hotspot residues, and query coverage, our modification, iBio-GATS, primarily focuses on hydrophobicity correspondence (HC) calculated in terms of SSD. In contrast to Bio-GATS, iBio-GATS omits other selection parameters due to limited data availability specific to iORs. The HC was calculated on the basis of helix definition present within the input Excel file as generated through AlignME (Section 3.2). User needs to enter the helix definitions to the provided input Excel file. Since *Mh*OR5 has three structures based on the same sequence, for ligand binding studies, 7LID (with bound eugenol) or 7LIG (with bound DEET) may be selected, to provide an appropriate ligand binding pocket.

### 3.4. Alignment Editing Using Mega11

The sequence alignments generated through AlignME might result in gaps within the helices. which can lead to helix deformation, rather than a shortened helix and consequently an inaccurate model. To remove gaps in the helices, manual alignment adjustments were done using MEGA (Molecular Evolutionary Genetics Analysis) 11 software [30].

### 3.5. Model Building Using Modeller 10.4 and AlphaFold2

The integration of the latest version, Modeller 10.4 [16] into the iBio-GATS workflow was achieved through shell scripting for building models for any iOR sequence. For validating the developed workflow, the structural models for *Mh*OR5, *Ab*Orco, *Dm*OR59b, and *Dmel*Orco were generated using iBio-GATS. Additionally, structural models for the same receptors were generated utilizing the current state-of-the-art artificial intelligence (AI) program AlphaFold2 [20]. The AlphaFold2 predicted model for the *Dm*OR59b sequence was downloaded from the AlphaFold database, using the Uniprot ID from https://alphafold.ebi.ac.uk/entry/Q9W1P8, accessed on 1 February 2024. As the structure of the *Dm*OR59b mutant sequence was not available in iORbase or AlphaFold2 databases, it was predicted using the AlphaFold2 available through Galaxy Australia [31]. The mutated *Dm*OR59b sequence was provided as an input to Alphafold2, and the top-ranked model was selected for further analysis. The same procedure is followed to obtain wild-type and mutant AlphaFold2 models of *Dmel*Orco (Uniprot ID: Q9VNB5).

### 3.6. Validation of the Predicted Model Structures

The accuracy of the predicted models, generated through iBio-GATS and Alphafold2, was evaluated by measuring the deviation from the template, expressed as root mean square deviation (RMSD). UCSF ChimeraX [12], a molecular structure visualization and analysis tool, was utilized to compute RMSD by superimposing the predicted structures and the template structure in Chimera’s matchmaker tool. To further assess the quality of predicted models, structure quality analysis was conducted using PSVS (Protein Structure Validation Software Suite) [32]. PSVS provides statistics based on the comparison between the given structure and experimental data. Additionally, the structure quality analysis of the predicted models was assessed using ProSA- web server [33].

### 3.7. Molecular Docking Studies

The ligand binding pockets for the 3D models were predicted through ICMpocketFinder embedded within ICM-pro 3.9 [35]. All molecular docking studies were performed through ICM-pro with the docking effort set to 3. DEET and VUAA1 structures were obtained from the PubChem database [https://pubchem.ncbi.nlm.nih.gov/, accessed on 25 January 2024]. The charges on the ligands were assigned through ICM with default parameters. The 2D ligand binding interactions were studied using BIOVIA Discovery Studio Visualizer (version 21.1) [40]. Further, ligand binding interactions were studied using UCSF ChimeraX 1.4 visualization tool [12].

### 3.8. Molecular Dynamics Studies

The structural models were embedded into the lipid bilayer composed of 42 POPE (1-palmitoyl-2-oleoyl-sn-glycero-3-phosphoethanolamine) and 22 POPC (1-palmitoyl-2-oleoyl-sn-glycero-3-phosphatidylcholine) molecules in each leaflet of the membrane using CHARMM-GUI server [41] based on the lipid composition in *Drosophila* membranes [42]. TIP3P water model was specified for the solvation of the system with an ionic concentration of 0.15 mM using NaCl. The Monte Carlo method was applied to place the ions. MD simulations were carried out through Amber 22 [36]. The average volume of the periodic boundary condition used in this study was around 656,679 Å^3^. Nose-Hoover thermostat in the NPT ensemble was used. The simulations of 100 ns were conducted under constant volume (NVE ensemble) without applying any pressure regulation or barostat. The membrane was equilibrated at constant pressure and the temperature was set to 298.15 K corresponding to the body temperature of *Drosophila melanogaster*. MD simulations were analysed through the R programming language [43] and graphed using the MD plot package [44]. Binding energy calculation was done using the molecular mechanics Poisson–Boltzmann surface area (MM/PBSA or PB) method, as this free energy prediction approach is more accurate than molecular docking scores and can be correlated to experimental values. This was performed using the CPPTRAJ [39] module of Amber 22.

## 4. Conclusions

This study is a novel approach for template selection for the structural modelling of insect odorant receptors, with low sequence identity, and few experimental structures from distant species. iBio-GATS, the semi-automated workflow developed based on this approach was successfully validated and applied to OR59b and Orco from *Drosophila melanogaster*. The workflow successfully generated high-quality models for *Dm*OR59b and *Dmel*Orco, supported by structure validation of the predicted models by structural quality analysis, docking studies, and molecular dynamics studies, concordant with experimental studies. Specifically, the iBio-GATS model generated a functional ligand binding site, supporting experimental polymorphism/mutational data, compared to models generated by AlphaFold2. This semi-automated workflow is generic and can be successfully applied in a high-throughput manner, to iOR sequences for any targeted insect pest genome. For future enhancements, the workflow could be updated by incorporating additional iOR templates as they become available. Also, it could be fully automated and integrated with OR-finding programs such as InsectOR to seamlessly use an insect’s complete genome sequence to get all iOR 3D models. 

## Figures and Tables

**Table 1:** SSD values for all helices, for the target, *Ab*Orco and the template, *Mh*OR5).

Helix No.	SSD	Helix No.	SSD
Helix 1	0.029	Helix 5	0.069
Helix 2	0.051	Helix 6	0.027
Helix 3	0.072	Helix 7	0.016
Helix 4	0.044		

**Table 2:** SSD values for helices of the target *Dm*OR59b sequence against both templates.

*Dm*OR59b Helix	Template: *Mh*OR5	Template: *Ab*Orco
Helix 1	0.219	0.113
Helix 2	0.040	0.085
Helix 3	0.158	0.179
Helix 4	0.177	0.151
Helix 5	0.026	0.116
Helix 6	0.040	0.031
Helix 7	0.173	0.182

**Table 3:** Structure quality analysis of Wt workflow and AlphaFold2 *Dm*Or59b models.

Structure Quality	WfWtOR59b Model	AfWtOR59b Model
Ramachandran plot: Favoured (%)	93.9	97.2
Ramachandran plot: Allowed (%)	6.0	2.8
Ramachandran plot: Disallowed (%)	0	0
RMSD for bond lengths	0.019 Å	0.013 Å
RMSD for bond angles	2.10°	1.70°
Procheck G factor (Z score)	1.48	3.02
ProSA-web (Z score)	−3.45	−6.02

**Table 4:** Insect odorant receptor (iOR)templates.

iOR	PDB ID	Organism	Bound Ligand	Resolution (Å)
*Ab*Orco	6C70	*Apocrypta bakeri*	none	3.5
*Mh*OR5	7LIC	*Machilis hrabei*	none	3.3
*Mh*OR5	7LID	*Machilis hrabei*	Eugenol	2.9
*Mh*OR5	7LIG	*Machilis hrabei*	DEET	2.9

## Data Availability

iBio-GATS scripts, along with the dependency requirements file, instructions for installation, and a detailed example file, are freely available to the scientific community from the GitHub repository (https://github.com/CVaans/iBio-GATS).

## Supplementary Materials

The following supporting information can be downloaded at: https://www.mdpi.com/article/10.3390/ijms25053055/s1.
