# Supplementary material for: iBio-GATS—A Semi-Automated Workflow for Structural Modelling of Insect Odorant Receptors
Source: Int J Mol Sci. 2024 Mar 6;25(5):3055. doi: 10.3390/ijms25053055 (PMC10932339; doi:10.3390/ijms25053055)
Supplement: Supplementary file 1 [file ijms-25-03055-s001.zip › ijms-2825836-supplementary.pdf]

iBio-GATS - a semi-automated workflow for structural modelling of insect odorant receptors

by Vaanathi Chidambara Thanu, Amara Jabeen, and Shoba Ranganathan

SUPPLEMENTARY FIGURES AND TABLES

Figure S1. Hydrophobicity plots for the *Ab*Orco sequence with the template *Mh*OR5

Figure S2. Helical wheel plots for the *Ab*Orco sequence with the template *Mh*OR5

Figure S3. iBio-GATS helix-wise alignment for the target sequence, *Ab*Orco with the template, *Mh*OR5

Figure S4. Full alignment for the target sequence, *Ab*Orco with the template, *Mh*OR5

Figure S5. Predicted models for *Ab*Orco and *Mh*OR5

Table S1. Helix definitions for *Dm*OR59b (UniProt ID: Q9W1P8)

Figure S6. Helical wheel plots for *Dm*OR59b compared with templates, *Mh*OR5 and *Ab*Orco

Figure S7. Final pairwise alignment of *Dm*OR59b with *Mh*OR5

Figure S8. Predicted models for wild *Dm*OR59b

Figure S9. Ramachandran plots

Figure S10. Binding pockets for predicted models of *Dm*OR59b with DEET

Figure S11. Final pairwise alignment of *Dmel*Orco with *Ab*Orco (PDB ID:6C70)

Table S2. Helix definitions for *Dmel*Orco (UniProt ID: Q9VNB5)

Figure S12. Hydrophobicity plots for the *Dmel*Orco sequence with templates *Ab*Orco and *Mh*OR5

Figure S13. Helical wheel plots for the *Dmel*Orco sequence compared templates, *Ab*Orco and *Mh*OR5

Figure S14. Structural overlay of predicted *Dmel*Orco model with its template *Ab*Orco.

Figure S15. RMSD plots of all four *Dmel*Orco complexes over 100ns of MD simulations

Figure S16. The structure-derived sequences of the template structures

Table S3. Helix positions for *Ab*Orco (PDB ID:6C70)

Table S4. Helix positions for *Mh*OR5 (PDB ID:7LIG).

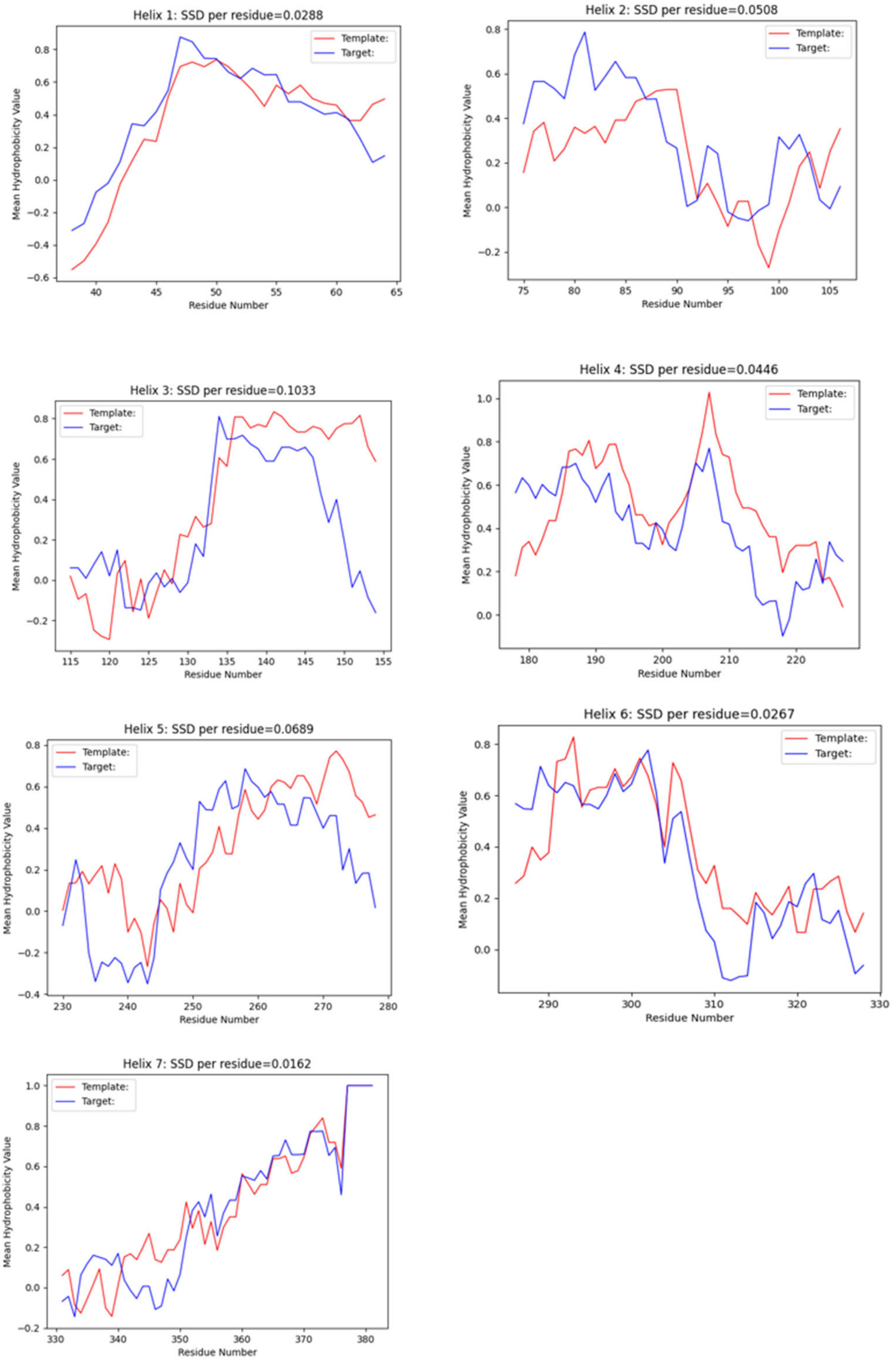

Figure S1. Hydrophobicity plots for the *Ab*Orco sequence with the template *Mh*OR5. Template PDB ID:7LIG .

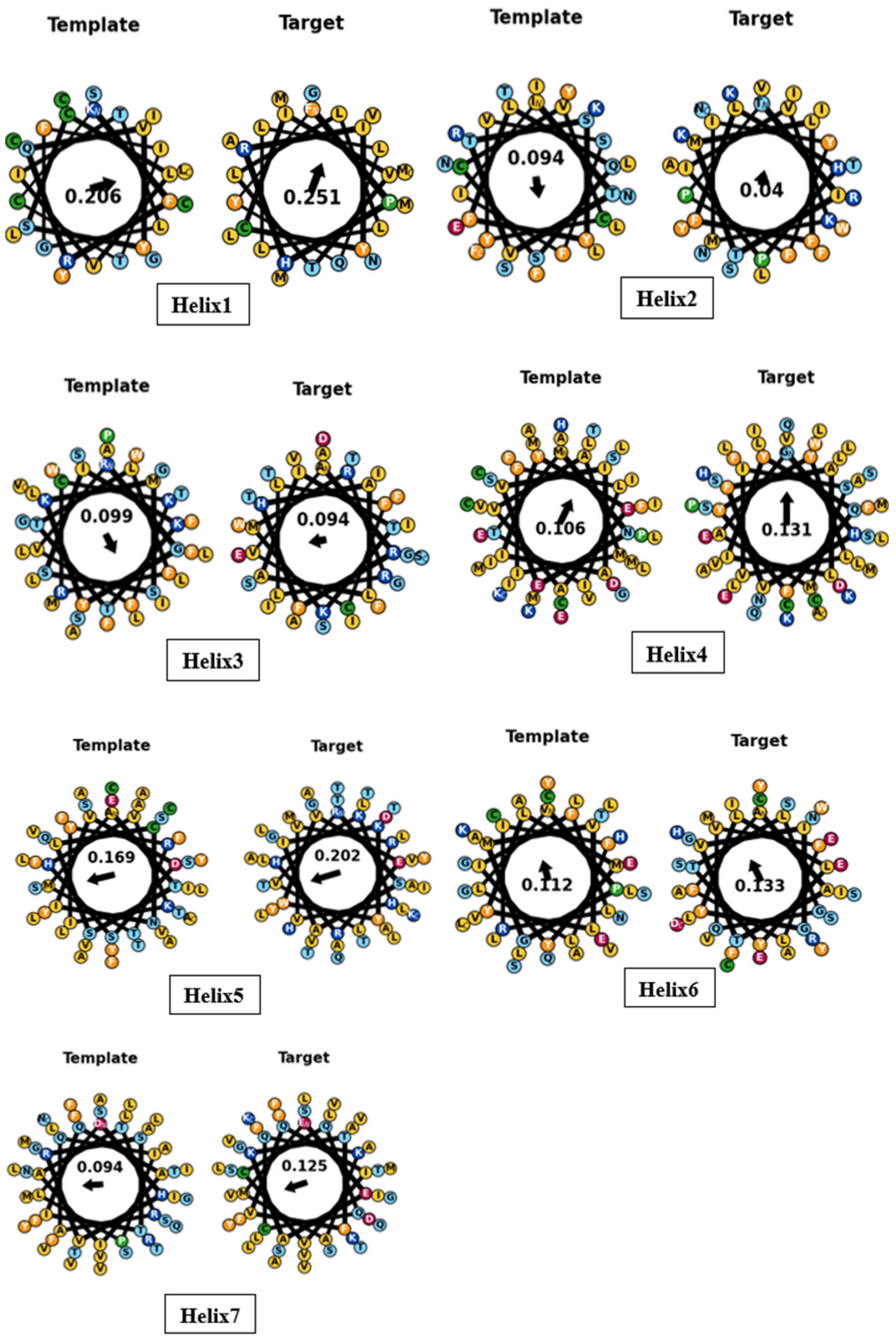

Figure S2. Helical wheel plots for the *Ab*Orco sequence with the template *Mh*OR5. Template PDB ID:7LIG.

```
Helix1
Template: KFRQVYSCLVITLGFITCSCYCIGLCL
Target:   FPHRIYCIVTLLLLLLQYGMMAVNLMM

Helix2
Template: ITVTSYFLQSCVCYVSFIINSRKLETLFNYLF-
Target:   TITMLFFLHPIVKMIYFPVRSKIFYKTLAIWNN

Helix3
Template: RGYKMSSIKTTLFRCKFVAFSLGILSFFGWLMWTLLPLAV
Target:   -ARFHALAITKMRRLLFCVAGATIFSVISWTGITFIEDS-

Helix4
Template: MNEVIAIYEAVAMIFLITAPMSSDIMFCVLMIFIVEHLKCLGMAIECTLK---
Target:   GHVFALIYQFYYLVISMAVSNSLDVLFCSWLLFACEQLQHLKAIMKPLMELSA

Helix5
Template: ATSLCNIVDSHVKIYRTMEIVQSVYSSYFATLFFTSCLAVCALAYFLAA
Target:   RSAIKYWVERHKHVVRLVTAVGDAYGVALLLHMLTTTITLTLLAYQATK

Helix6
Template: VPGMVLYLMYIFLRIFLLCLLATEVAEQGLNLCHAGYSSKLVL
Target:   AATVIGYLLYTLGQVFLFCIFGNRLIEESSSVMEAAYSCHWYD

Helix7
Template: DHVRSTIQAIATRAQIPLSITGARFFTVNLSFLASMAGVMLTYFIVLLQVN
Target:   EEAKTFVQIVCQQCQKAMSISGAKFFTVSLDLFASVLGAVVTYFMVLVQLK
```

Figure S3. iBio-GATS helix-wise alignment for the *Ab*Orco target, with the, *Mh*OR5 template.

```
>P1;Template
structureX:7LIG:: :: ::: 0.00: 0.00
DDYIHLR-KWI-K-RIGIILRISGHW--PF--R-LPHEK--R---N---Q-HKSKFRQVYSCLVITLGFITCSCYCIGLC
LSES--IAQALN---NITVTSYFLQSCVCYVSFIINSRKLETLFNYLF-ENEVVGC-P------RGYKMSSIKTTLFRCK
FVAFSLGILSFFGWLMWTLLPLAVLV--V-D-QT-S--L----RF--VEAW-YPFDT-TTSPMNEVIAIYEAVAMIFLIT
APMSSDIMFCVLMIFIVEHLKCLGMAIECTLK------------G-----------------------------------
----D------------------------------ATSLCNIVDSHVKIYRTMEIVQSVYSSYFATLFFTSCLAVCALAY
FLAATSTS---FTRVPGMVLYLMYIFLRIFLLCLLATEVAEQGLNLCHAGYSSKLVLASDHVRSTIQAIATRAQIPLSIT
GARFFTVNLSFLASMAGVMLTYFIVLLQVN*
>P1;Target
sequence:6C70:: :: ::: 0.00: 0.00
---GPGRAK--FKHQ-G--L-V-AD-LLP-NIRVM-QG-VGHFMFNYYSEGKK-FPHRIYCIVTLLLLLLQYGMMAVNLM
M-ESDDV-D--DLTANTITMLFFLHPIVKMIYFPVRSKIFYKTLAIWNNPN---S-HPLFAESN-ARFHALAITKMRRLL
FCVAGATIFSVISWTGITFIEDS--VKRITDPETNETTIIPIPR-LMIRT-FYPFNAMSGA-GHVFALIYQFYYLVISMA
VSNSLDVLFCSWLLFACEQLQHLKAIMKPLMELSATLDTVVPNSGELFKAGSADHLRESQGVQPSGNGDNVLDVDLRGIY
SNRQDFTATFRPTAGTTFNGGVGPNGLTKKQEMLVRSAIKYWVERHKHVVRLVTAVGDAYGVALLLHMLTTTITLTLLAY
QATK-VNGVNVY--AATVIGYLLYTLGQVFLFCIFGNRLIEESSSVMEAAYSCHWYDGSEEAKTFVQIVCQQCQKAMSIS
GAKFFTVSLDLFASVLGAVVTYFMVLVQLK*
```

Figure S4. Full alignment for the *Ab*Orco sequence, with the *Mh*OR5 template.

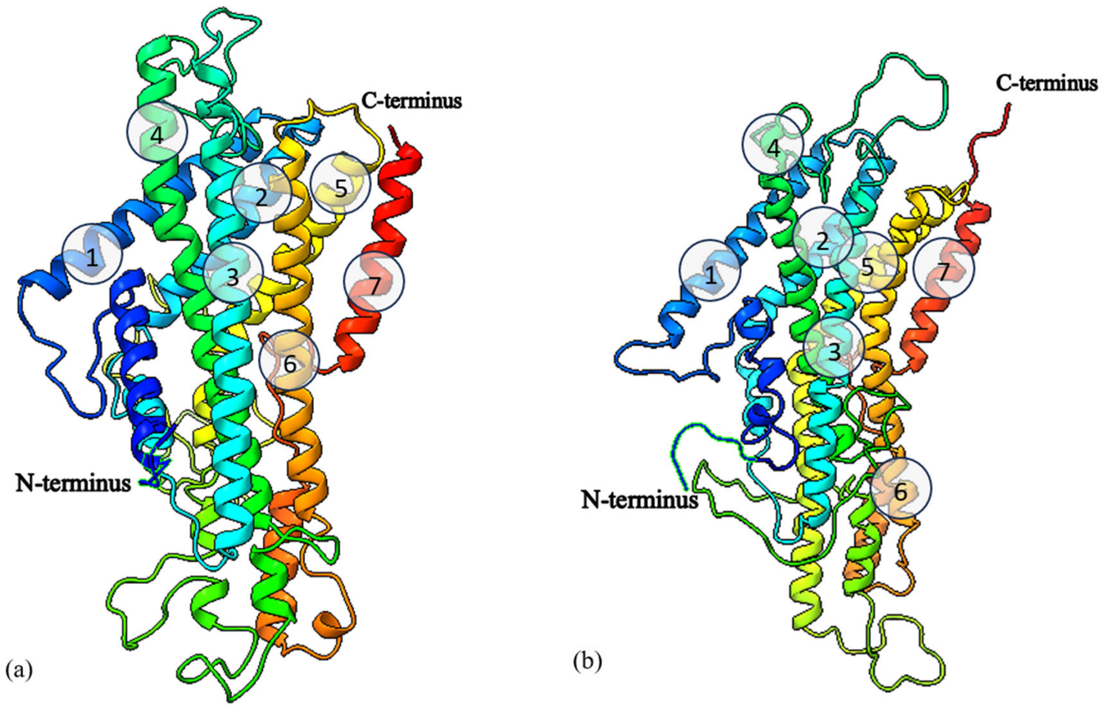

Figure S5. Predicted models for *Ab*Orco and *Mh*OR5. (a) The model predicted for *Ab*Orco sequence using the template *Mh*OR5 and (b) the model predicted for *Mh*OR5 using the template *Ab*Orco. The seven helices, C-terminus and N-terminus are labelled.

Table S1. Helix definitions for *Dm*OR59b (UniProt ID: Q9W1P8)

Helix	Helix start	Helix end	Centre residue
1	47	73	Y60
2	88	119	F104
3	121	160	S146
4	183	232	D206
5	242	290	S267
6	297	339	L321
7	342	392	F367

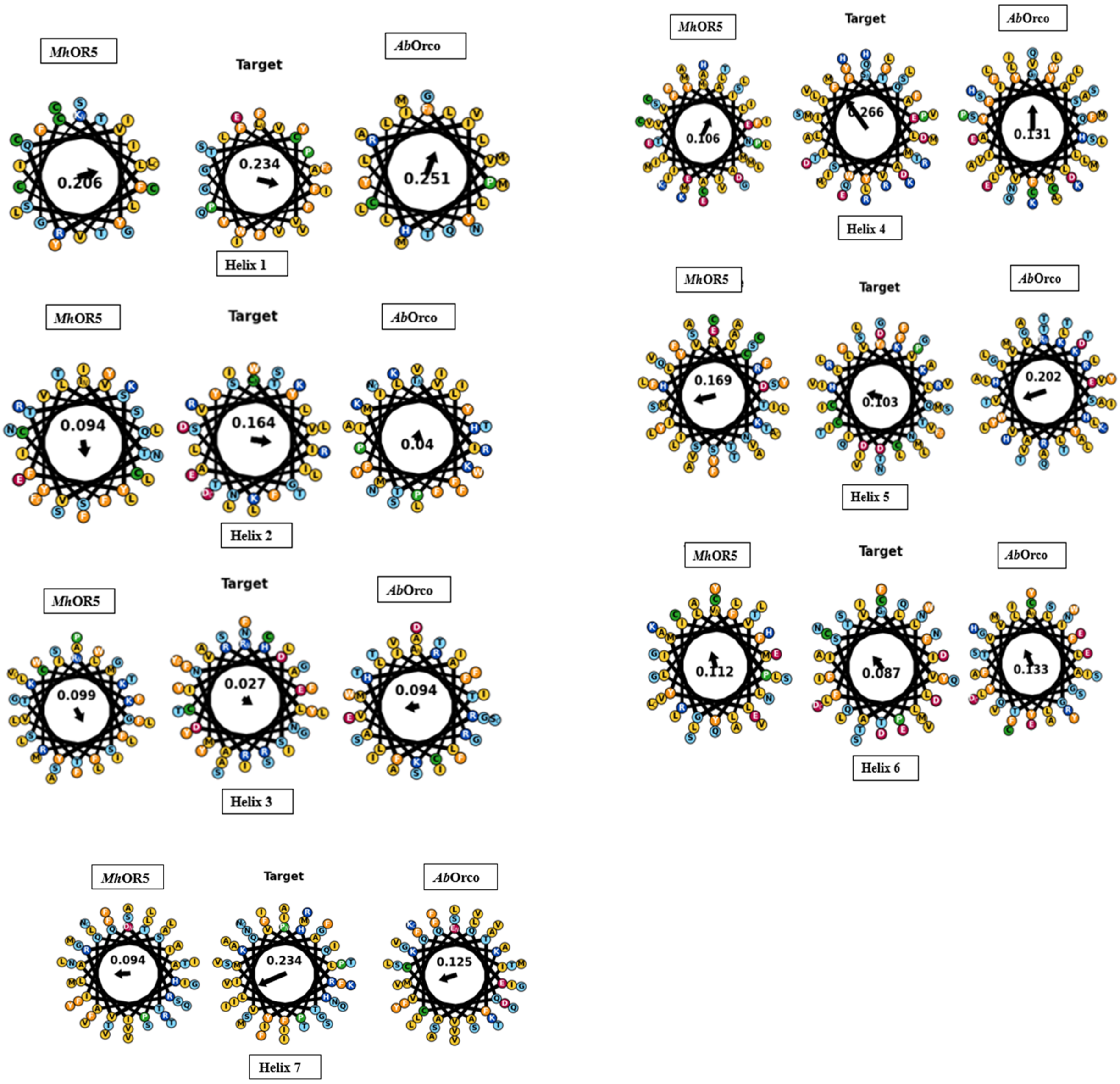

Figure S6. Helical wheel plots for *Dm*OR59b compared with templates, *Mh*OR5 and *Ab*Orco. The helical moments of *Dm*OR59b are better aligned to those of *Mh*OR5 than those of *Ab*Orco.

```
                         10        20        30        40        50  He1   60
7LIG_1Chain_A    ------------DDYIHLRKWIKRIGIILRISGHWPFRLPHEKRNQHKSKFRQVYSCLVI
NP_523822.1od    MAVFKLIKPAPLTEKVQSRQGNIYLYRAMWLIGWIP---PKEGVLRYVYLFWTCVPFAFG

                         70        80        90       100  He2  110       120
7LIG_1Chain_A    TLGFITCSCYCIGLCLS----ESIAQALNNITVTSYFLQSCVCYVSFIINSRKLETLFNY
NP_523822.1od    VFYLPVGFIISYVQEFKNFTPGEFLTSLQVCINVYGASVKSTITYLFLWRLRKTEILLDS

                        130       140       150  He3  160       170       180
7LIG_1Chain_A    LFENEVVGCPRGYKMSSIKTTLFRCKFVAFSLGILSFFGWLMWTLLPLAVLVVDQTSLRF
NP_523822.1od    LDK-------RLANDSDRERIHNMVARCNYAFLIYSFIYCGYAGSTFLSYALSGRPPWSV

                        190       200  He4  210       220       230       240
7LIG_1Chain_A    VEAWYPFDTTTSPMNEVIAIYEAVAMIFLITAPMSSDIMFCVLMIFIVEHLKCLGMAIEC
NP_523822.1od    YNPFIDWR-DGMGSLWIQAIFEYITMSFAVLQDQLSDTYPLMFTIMFRAHMEVLKDHVRS

                        250       260       270  He5  280       290       300
7LIG_1Chain_A    TLKGD-------ATSLCNIVDSHVKIYRTMEIVQSVYSSYFATLFFTSCLAVCALAYFLA
NP_523822.1od    LRMDPERSEADNYQDLVNCVLDHKTILKCCDMIRPMISRTIFVQFALIGSVLGLTLVNVF

                        310       320       330  He6  340       350       360
7LIG_1Chain_A    ATSTSFTRVPGMVLYLMYIFLRIFLLCLLATEVAEQGLNLCHAGYSSKLVLASDHVRSTI
NP_523822.1od    FFS-NFWKGVASLLFVITILLQTFPFCYTCNMLIDDAQDLSNEIFQSNWVDAEPRYKATL

                        370       380       390  He7  400       410
7LIG_1Chain_A    QAIATRAQIPLSITGARFFTVNLSFLASMAGVMLTYFIVLLQVN------
NP_523822.1od    VLFMHHVQQPIIFIAGGIFPISMNSNITVAKFAFSIITIVRQMNLAEQFQ
```

Figure S7. Final pairwise alignment of *Dm*OR59b with *Mh*OR5. The AlignMe 1.2 output was edited manually to remove gaps in the helical regions using the MEGA 11 sequence viewer.

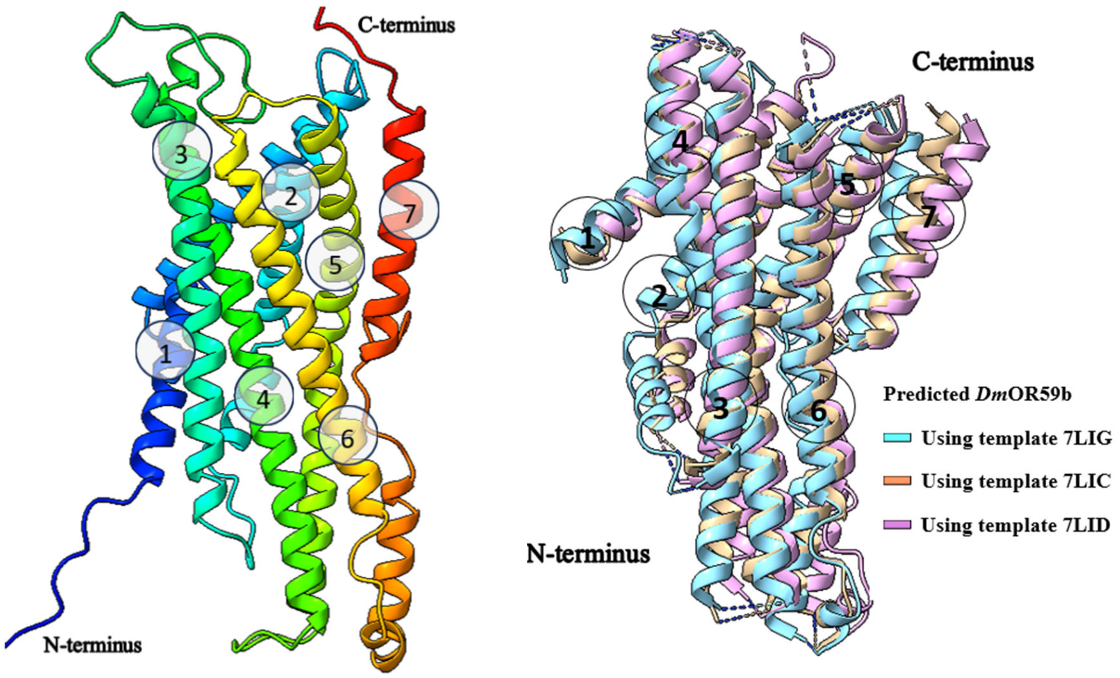

Figure S8. Predicted models for *Dm*OR59b sequence. (a) The top predicted model for *Dm*OR59b (WfWtOR59b) using the template *Mh*OR5 (PDB:7LIG). (b) Structural overlay of predicted models (showing only helices) of *Dm*OR59b (WfWtOR59b) using all three templates of *Mh*OR5. The seven helices are labelled.

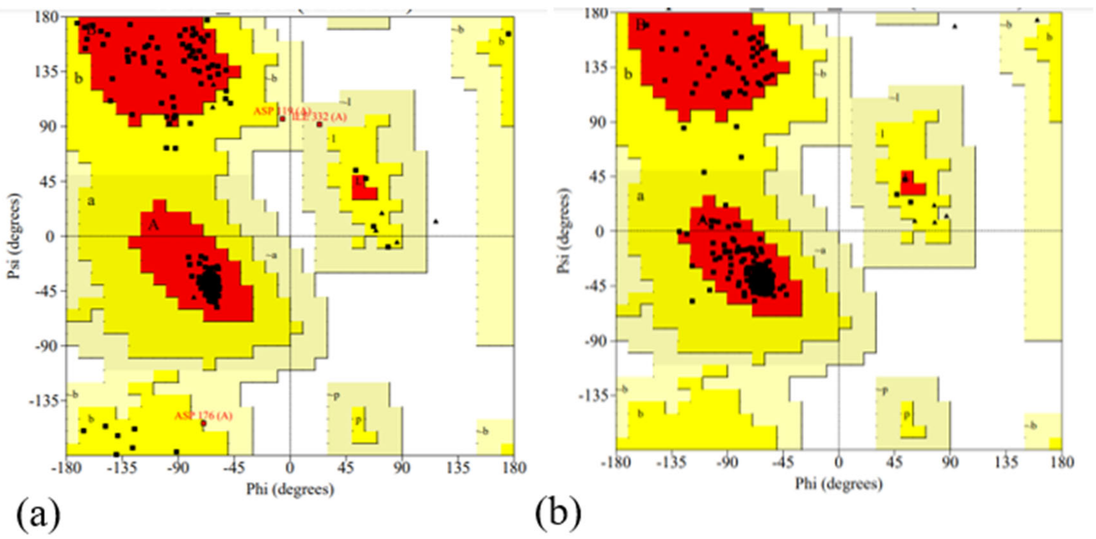

Figure S9. Ramachandran plots for *Dm*OR59b. (a) iBio-GATS workflow model, WfWtOR59b and (b) AlphaFold2 model, AfWtOR59b.

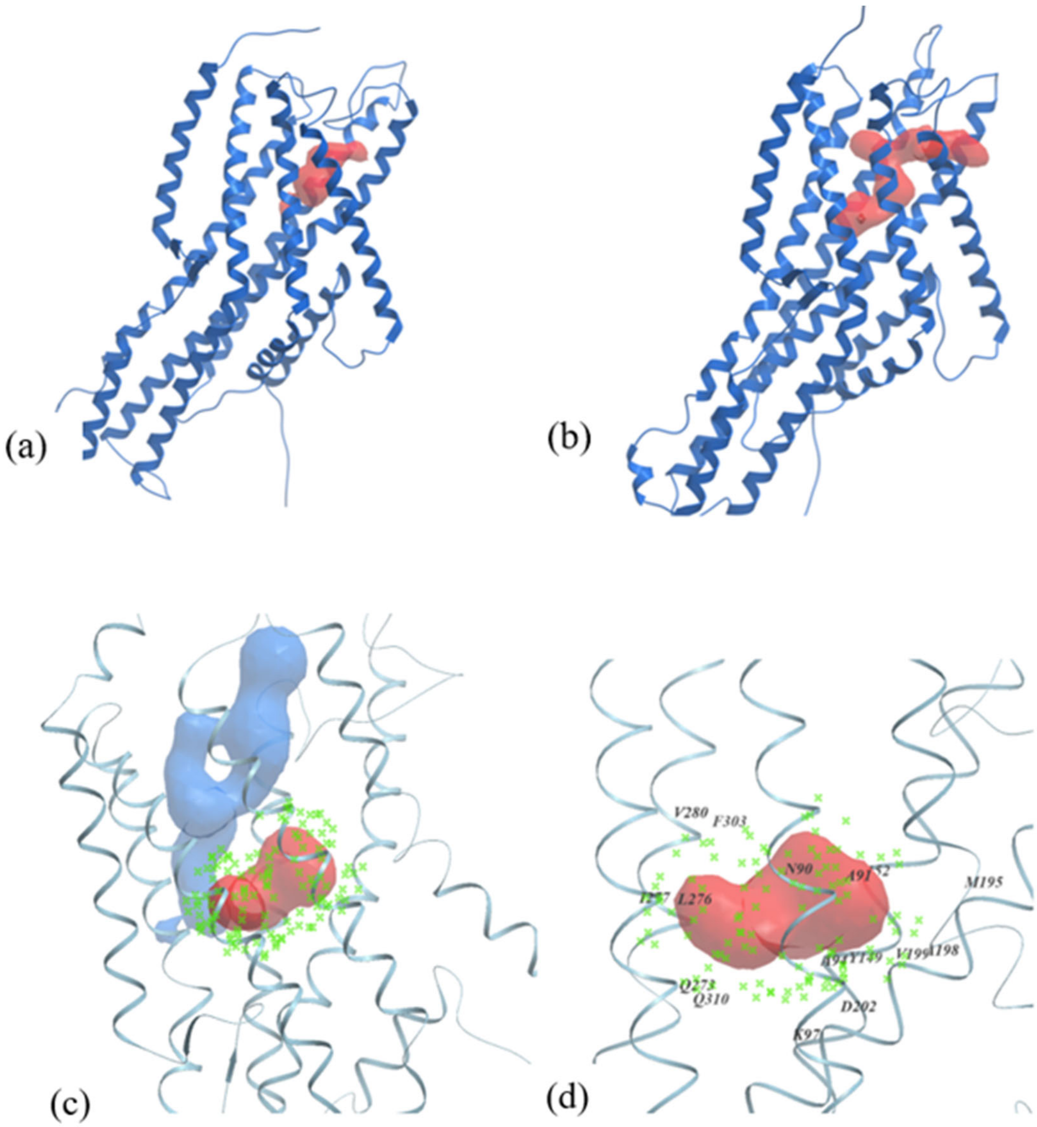

Figure S10. Binding pockets for predicted models of *Dm*OR59b with DEET

The ICM binding pockets of the workflow (Wf) and Alpha Fold (Af) wild type (Wt) and mutant (Mt) models of *Dm*OR59b. (a) WfWtOR59b, (b) WfMtOR59b, (c) AfWtOR59b and (d). AfMtOR59b.

```
                   10        20        30        40        50   He1  60
6C70_1ChainsA   -------FKHQGLVADLLPNIRVMQGVGHFMFNYYSEGKKFPHRIYCIVTLLLLLLQYGM
spQ9VNB5ORCO_   MTTSMQPSKYTGLVADLMPNIRAMKYSGLFMHNF-TGGSAFMKKVYSSVHLVFLLMQFTF
                   70        80   He2  90       100       110       120
6C70_1ChainsA   MAVNLMMESDDVDDLTANTITMLFFLHPIVKMIYFPVRSKIFYKTLAIWNNPNSHPLFAE
spQ9VNB5ORCO_   ILVNMALNAEEVNELSGNTITTLFFTHCITKFIYLAVNQKNFYRTLNIWNQVNTHPLFAE
                  130       140   He3 150       160       170       180
6C70_1ChainsA   SNARFHALAITKMRRLLFCVAGATIFSVISWTGITFIEDS---------------PIPRL
spQ9VNB5ORCO_   SDARYHSIALAKMRKLFFLVMLTTVASATAWTTITFFGDSVKMVVDHETNSSIPVEIPRL
                  190       200   He4 210       220       230       240
6C70_1ChainsA   MIRTFYPFNAMSGAGHVFALIYQFYYLVISMAVSNSLDVLFCSWLLFACEQLQHLKAIMK
spQ9VNB5ORCO_   PIKSFYPWNASHGMFYMISFAFQIYYVLFSMIHSNLCDVMFCSWLIFACEQLQHLKGIMK
                  250       260       270       280       290       300
6C70_1ChainsA   PLMELSAT----------------------------------------------------
spQ9VNB5ORCO_   PLMELSASLDTYRPNSAALFRSLSANSKSELIHNEEKDPGTDMDMSGIYSSKADWGAQFR
                  310       320       330  He5  340       350       360
6C70_1ChainsA   -------------------------GLTKKQEMLVRSAIKYWVERHKHVVRLVTAVGDAY
spQ9VNB5ORCO_   APSTLQSFGGNGGGGNGLVNGANPNGLTKKQEMMVRSAIKYWVERHKHVVRLVAAIGDTY
                  370       380       390  He6  400       410       420
6C70_1ChainsA   GVALLLHMLTTTITLTLLAYQATKVNGVNVYAATVIGYLLYTLGQVFLFCIFGNRLIEES
spQ9VNB5ORCO_   GAALLLHMLTSTIKLTLLAYQATKINGVNVYAFTVVGYLGYALAQVFHFCIFGNRLIEES
                  430       440       450  He7  460       470       480
6C70_1ChainsA   SSVMEAAYSCHWYDGSEEAKTFVQIVCQQCQKAMSISGAKFFTVSLDLFASVLGAVVTYF
spQ9VNB5ORCO_   SSVMEAAYSCHWYDGSEEAKTFVQIVCQQCQKAMSISGAKFFTVSLDLFASVLGAVVTYF
                487
6C70_1ChainsA   MVLVQLK
spQ9VNB5ORCO_   MVLVQLK
```

Figure S11. Final pairwise alignment of *Dmel*Orco with *Ab*Orco (PDB ID:6C70) obtained from AlignMe 1.2. The sequence identity between the two sequence is 55.2%.

Table S2. Helix definitions for *Dmel*Orco (UniProt ID: Q9VNB5)

Helix	Helix start	Helix end	Centre residue
1	40	66	L54
2	78	110	L94
3	122	159	S146
4	194	246	D217
5	335	383	G260
6	391	433	L415
7	436	486	F461

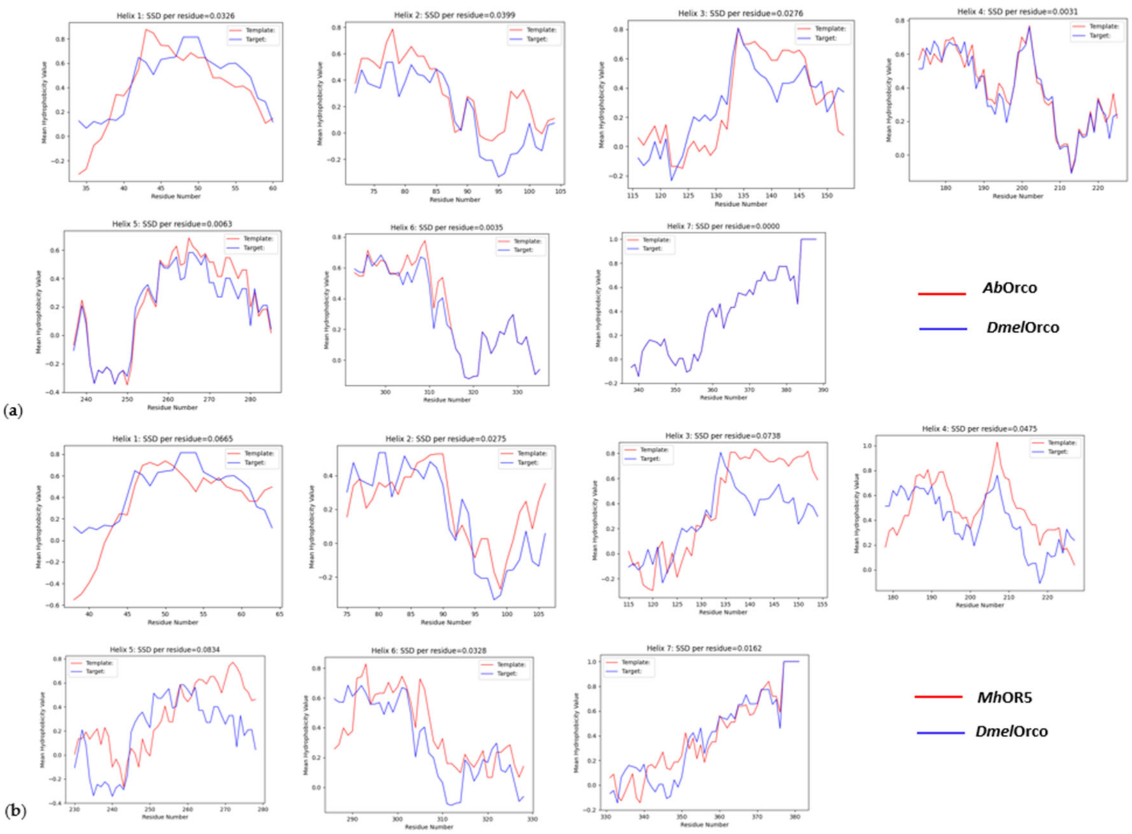

Figure S12. The result summary output from workflow shows the hydrophobicity plots for all the seven helices for *Dmel*Orco compared with both the templates, *Ab*Orco and *Mh*OR5

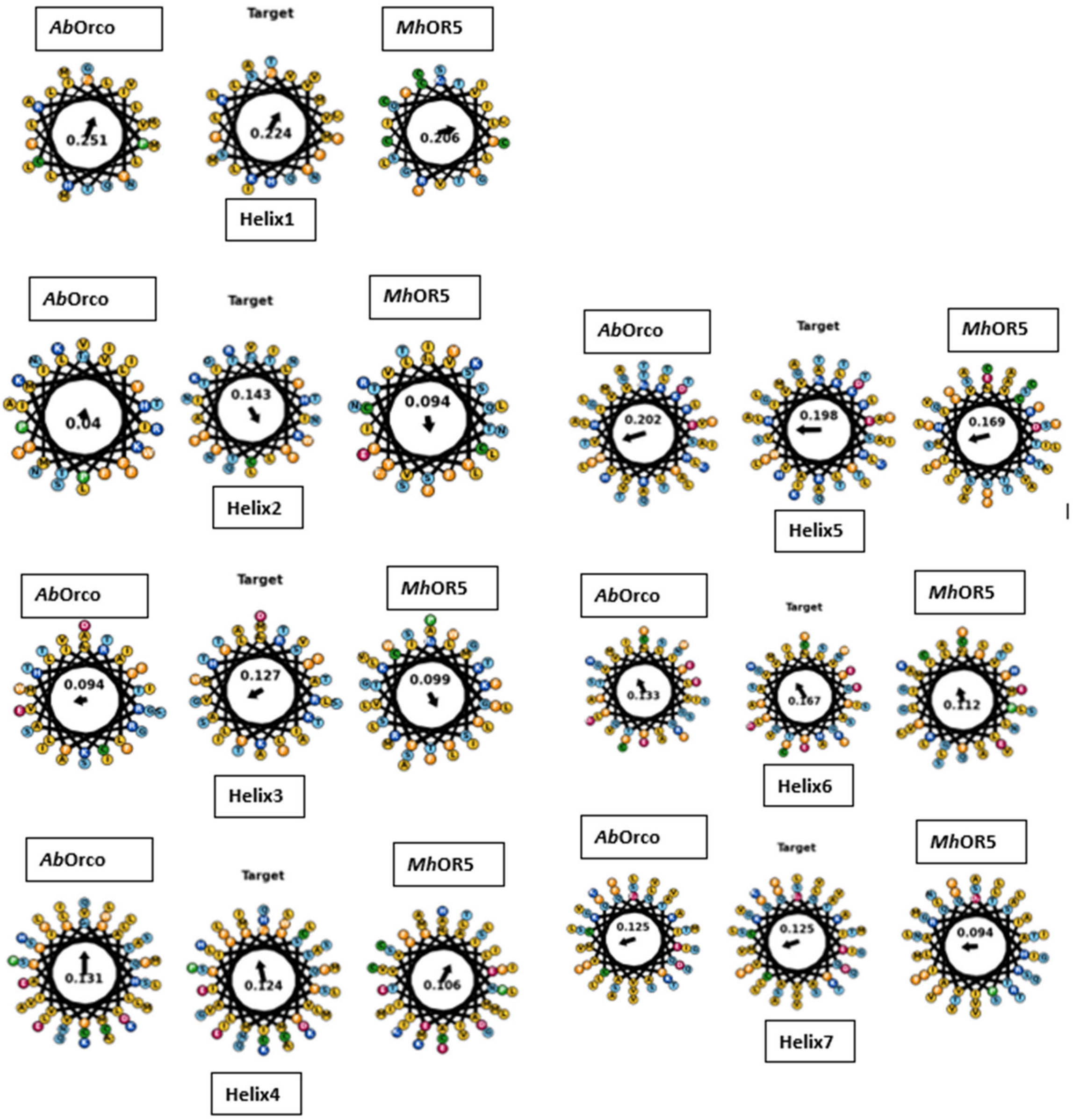

Figure S13. The result summary output from workflow shows the helical plots for all the seven helices for *Dmel*Orco compared with both the templates, *Ab*Orco and *Mh*OR5. The helical moments of *Dmel*Orco are better aligned to those of *Ab*Orco than those of *Mh*OR5.

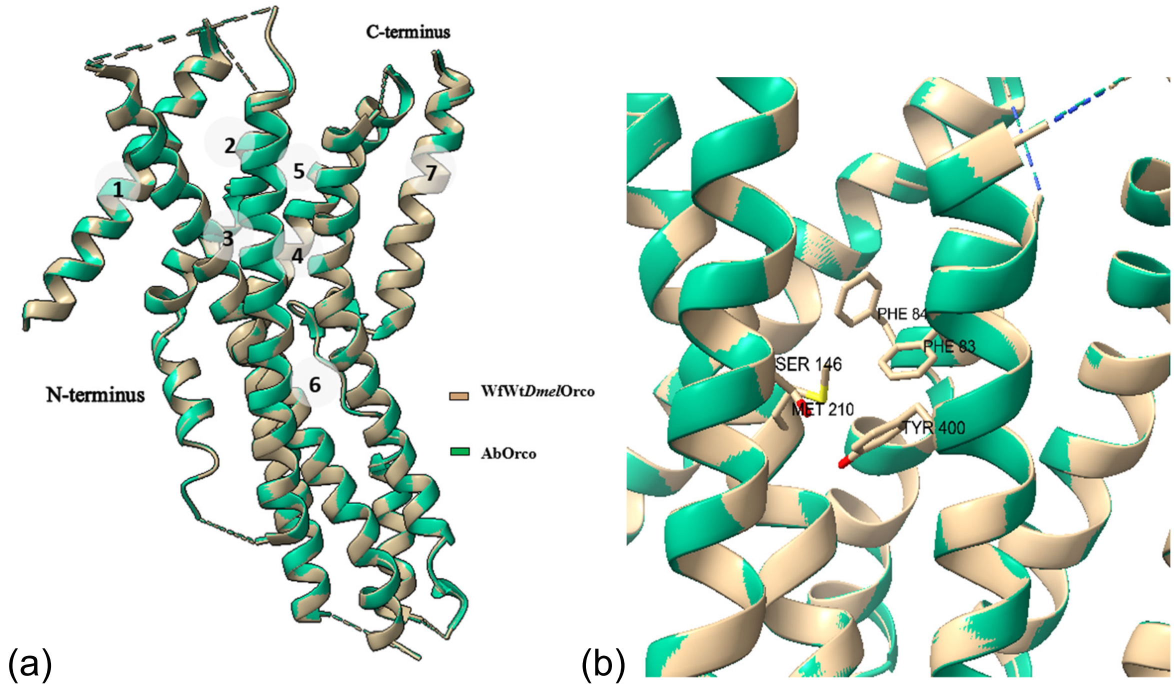

Figure S14. Structural overlay of predicted WfWt*Dmel*Orco model with its template *Ab*Orco. (PDB ID:6C70). (a) Superposed helical regions, with an RMSD of 0.1 Å, and the seven helices numbered. (b) The five residues (F83, F84, M210, S146, Y400) that were frequently identified to interact with VUAA1 in the binding site are captured.

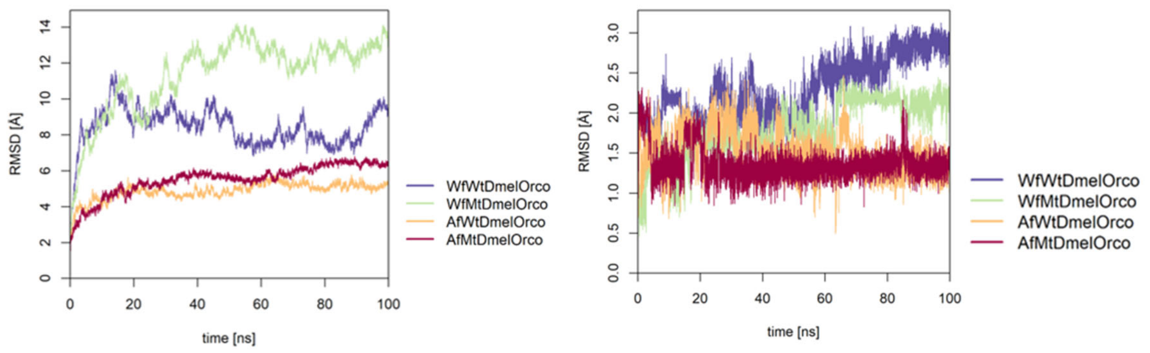

Figure S15. RMSD plots of all four *Dmel*Orco complexes over 100 ns of MD simulations. (a) *Dmel*Orco complex with bound VUAA1 and (b) the ligand VUAA1 alone.

```
>7LIG|Chains A|MhOR5|Machilis hrabei (438506)
DDYIHLRKWIKRIGIILRISGHWPFRLPHEKRNQHKSKFRQVYSCLVITLGFITCSCYCIGLCLSESIAQALNNITVTS
YFLQSCVCYVSFIINSRKLETLFNYLFENEVVGCPRGYKMSSIKTTLFRCKFVAFSLGILSFFGWLMWTLLPLAVLVVD
QTSLRFVEAWYPFDTTTSPMNEVIAIYEAVAMIFLITAPMSSDIMFCVLMIFIVEHLKCLGMAIECTLKGDATSLCNIV
DSHVKIYRTMEIVQSVYSSYFATLFFTSCLAVCALAYFLAATSTSFTRVPGMVLYLMYIFLRIFLLCLLATEVAEQGLN
LCHAGYSSKLVLASDHVRSTIQAIATRAQIPLSITGARFFTVNLSFLASMAGVMLTYFIVLLQVN
>6C70|Chains A|Odorant receptor|Apocrypta bakeri (490712)
FKHQGLVADLLPNIRVMQGVGHFMFNYYSEGKKFPHRIYCIVTLLLLLLQYGMMAVNLMMESDDVDDLTANTITMLFFL
HPIVKMIYFPVRSKIFYKTLAIWNNPNSHPLFAESNARFHALAITKMRRLLFCVAGATIFSVISWTGITFIEDSPIPRL
MIRTFYPFNAMSGAGHVFALIYQFYYLVISMAVSNSLDVLFCSWLLFACEQLQHLKAIMKPLMELSATGLTKKQEMLVR
SAIKYWVERHKHVVRLVTAVGDAYGVALLLHMLTTTITLTLLAYQATKVNGVNVYAATVIGYLLYTLGQVFLFCIFGNR
LIEESSSVMEAAYSCHWYDGSEEAKTFVQIVCQQCQKAMSISGAKFFTVSLDLFASVLGAVVTYFMVLVQLK
```

Figure S16. The structure-derived sequences of the template structures. *Mh*OR5: PDB ID: 7LIG and *Ab*Orco: PDB ID: 6C70.

Table S3. Helix positions for *Ab*Orco (PDB ID:6C70)

Helix	Helix start	Helix end	Centre residue
1	34	60	L47
2	72	104	F88
3	116	153	S140
4	173	225	D196
5	237	285	G262
6	293	335	L317
7	338	388	F363

Table S4. Helix positions for *Mh*OR5 (PDB ID:7LIG)

Helix	Helix start	Helix end	Centre residue
1	38	64	G51
2	75	106	F91
3	115	154	S140
4	178	227	D201
5	230	278	S255
6	286	328	V310
7	331	381	F356
